# Summarizing the extent of visit irregularity in longitudinal data

**DOI:** 10.1186/s12874-020-01023-w

**Published:** 2020-05-29

**Authors:** Armend Lokku, Lily S. Lim, Catherine S. Birken, Eleanor M. Pullenayegum, on behalf of the TARGet Kids! Collaboration

**Affiliations:** 1 Child Health Evaluative Sciences, Hospital for Sick Children, Toronto, Ontario Canada; 2 Dalla Lana School of Public Health, University of Toronto, 155 College Street, Toronto, Ontario M5T 3M7 Canada; 3 Department of Pediatrics, University of Manitoba, Winnipeg, Manitoba Canada; 4 Children’s Hospital Research Institute of Manitoba, University of Manitoba, Winnipeg, Canada; 5 Division of Pediatric Medicine and the Pediatric Outcomes Research Team (PORT), Hospital for Sick Children, Toronto, Ontario Canada; 6 Sick Kids Research Institute, Toronto, Ontario Canada; 7 Institute of Health Policy, Management, and Evaluation, University of Toronto, Toronto, Ontario Canada; 8 Department of Pediatrics, Faculty of Medicine, University of Toronto, Toronto, Ontario Canada

**Keywords:** Longitudinal data, Irregular visits, Visit process, Missing data mechanism, Visit intensity

## Abstract

**Background:**

Observational longitudinal data often feature irregular, informative visit times. We propose descriptive measures to quantify the extent of irregularity to select an appropriate analytic outcome approach.

**Methods:**

We divided the study period into bins and calculated the mean proportions of individuals with 0, 1, and > 1 visits per bin. Perfect repeated measures features everyone with 1 visit per bin. Missingness leads to individuals with 0 visits per bin while irregularity leads to individuals with > 1 visit per bin. We applied these methods to: 1) the TARGet Kids! study, which invites participation at ages 2, 4, 6, 9, 12, 15, 18, 24 months, and 2) the childhood-onset Systemic Lupus Erythematosus (cSLE) study which recommended at least 1 visit every 6 months.

**Results:**

The mean proportions of 0 and > 1 visits per bin were above 0.67 and below 0.03 respectively in the TARGet Kids! study, suggesting repeated measures with missingness. For the cSLE study, bin widths of 6 months yielded mean proportions of 1 and > 1 visits per bin of 0.39, suggesting irregular visits.

**Conclusions:**

Our methods describe the extent of irregularity and help distinguish between protocol-driven visits and irregular visits. This is an important step in choosing an analytic strategy for the outcome.

## Background

Observational longitudinal data often feature visit times that vary across individuals with the potential for the timings and frequency of visits to be associated with the study outcome. Visit irregularity can lead to misleading conclusions [1] and should therefore be accounted for in analyses of the outcome trajectory [2]. For example, in a randomized trial of the interventions to reduce homelessness, individuals with greater levels of homelessness were likely to visit more frequently [1]. When the visit process was ignored the group receiving a case manager only had 0.71% more days homeless than the standard care; when the visit process was accounted for the effect estimate reversed direction with the case manager group having 1.64% fewer days homeless. In another example, Buzkova et al [3] estimated the prevalence of pneumonia amongst Kenyan mothers with HIV-1 to be 2.89% when the visit process was ignored; the estimate almost halved to 1.48% after accounting for visits. Observational data are readily available (e.g. in administrative databases, electronic medical records); however, data collected over the course of usual care are particularly liable to irregular visiting patterns.

The problem of visit irregularity is analogous to missing data. The key difference between irregular data and missing data is that the latter occurs when a scheduled measurement is not recorded, whereas irregular data describes the presence of imbalanced visiting patterns across individuals, often in the absence of a study wide follow-up schedule. In statistical terms, data is missing when visit times are fixed by design and whether the visit occurs is a random variable. With irregular visits, the timings of visits are the random variables.

The possibility for biased results in the presence of missing data is generally recognized in applied settings [4], and this consensus has led to the exploration of missing data patterns being recommended (e.g. STROBE, CONSORT 2010) [5, 6]. Summarizing missing data typically begins by recording the frequency (or percentage) of individuals with missing values for each variable (STROBE [5]), upon which the severity of the problem can be judged. For example, if the data is judged to be missing at random (or completely at random), one might proceed with techniques that deal with missing longitudinal data such as multiple imputation [7] or inverse-probability weighting [8]. On the other hand, unless missingness is known to be completely at random, missing values may render further analysis futile as informative missingness can lead to bias as missingness increases.

Given that irregularity can lead to bias, irregular data should be explored with the same rigor as is done with missing data. Irregularity exists on a continuum where on one extreme the extent of irregularity can vary to the point where no two individuals share the same visit times. At the other extreme, visit times can resemble perfect repeated measures where every individual has 1 visit at each pre-specified visit time in the protocol. In practice, there are scenarios between both extremes where visits are intended to be repeated measures but the timings of scheduled visits vary across individuals, scheduled visits are missing, or there are unscheduled visits. There are different techniques for analyzing irregular data versus repeated measures, but it can be difficult to decide at what point the data should no longer be treated as repeated measures, but as irregular data. Farzanfar et al [9] performed a systematic review of longitudinal studies to explore how irregularity is reported and handled in practice. They observed that of the 44 eligible studies: 86% of the studies did not report enough information to assess if it was necessary to account for informative visit timings, 3 studies reported on the gaps between visits, 2 studies assessed predictors of visit times, and only 1 study used a specialized method for irregular longitudinal data. One of the reasons why visit irregularity is ignored in practice is that most of the literature on this topic is highly technical.

There are currently no proposed measures for quantifying the extent of irregularity in longitudinal data. This paper provides intuitive visual measures that can be used by researchers who are not experts in statistics along with the respective R code to implement these measures in practice. This paper demonstrates how these descriptive measures can help distinguish between individually-driven irregular visits and protocol-driven regular visits, and illustrates how to examine the underlying visit process to select an appropriate statistical approach for the outcome.

## Methods

### Datasets

We will illustrate our proposed measures of irregularity with the following two datasets.

#### Pre-specified visit times: TARGet kids!

The TARGet Kids! study enrolls healthy children aged 0–5 years and follows them until age 18, with the aim of investigating the relationship between early life exposures and later health problems including obesity, micronutrient deficiencies, and developmental problems [10]. Well-child visits are recommended at ages 2, 4, 6, 9, 12, 15, 18, 24 months, and then every 12 months afterwards, with vaccinations occurring at ages 2, 4, 6, 12, 15, 18 months. Parents also bring their children for “sick” visits as needed. Individuals are recruited and enrolled by research assistants who approach them at well-child visits. In general, most well-child visits did not occur prior to the expected visit schedule because the physician could not bill for an early visit, and vaccinations could only occur once a child reaches a specific age. For example, the Measles, Mumps and Rubella (MMR) vaccine cannot be administered until a child is 12 months old.

#### No pre-specified visit times: child systemic lupus Erythematosus study

The child lupus study was a retrospective inception cohort study of patients who were diagnosed with childhood-onset Systemic Lupus Erythematosus (cSLE) and followed at a single center with a dedicated cSLE clinic. This cohort was followed from childhood into adulthood. Visit dates ranged from January 1st, 1985 to September 30th, 2011. Individuals are followed at least once every 6 months; however, visit frequency depended on the severity of their disease. The primary objective of this study was to assess differences in disease activity trajectories among all cSLE patients.

### Measures for quantifying the extent of visit irregularity

The following measures can be used to assess the extent of visit irregularity and help inform the modelling approach for the outcome. They can also help determine whether observed visits can be viewed as repeated measures subject to missingness. The proposed measures are based on techniques used to explore missing data. In a repeated measures design, summarizing missing values begins by recording the percentage of missing values at each pre-specified visit time. In addition, predictors of being observed at a pre-specified visit time can be evaluated using a regression model (e.g. logistic regression). We adapt these concepts to the context of irregular data. We consider studies with pre-specified visit times in the protocol, and studies which do not pre-specify visit times in the protocol.

#### Pre-specified visit times

**Fig. 1:**
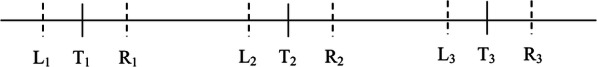
Specifying bin widths for pre-specified visit times

We propose constructing bins around pre-specified visit times. Let the time frame of interest be (0, τ), and let T_j_ denote the j^th^ pre-specified visit time (j = 1, 2,...k). The j^th^ bin is given by the interval (L_j_, R_j_), where L_j_ and R_j_ are chosen to specify the left and right cut-points of the j^th^ bin respectively (Fig. 1). We require that R_j_ < L_j + 1_ (for all values of j) so that bins do not overlap, and that L_j_ < T_j_ < R_j_. These bins can be used to calculate summary statistics such as the proportions of individuals with 0, 1, and > 1 visits per bin.

Bin widths should be specified according to clinical context as appropriate. For example, the HbA1C blood test measures blood glucose levels from the previous 3 months (levels are known to be stable during this time period [11]), and hence bin widths should not be less than this. Bins can have different widths across the study period to account for known patterns in visit intensity (e.g. more frequent visits in the winter). Another approach to specifying bin widths is to use the percentage of the time gap between the pre-specified visit times (T_j_). For example, 10% of the gap implies that L_j_ = T_j_ - 0.1(T_j_ – T_j-1_), and R_j_ = T_j_ + 0.1(T_j + 1_ – T_j_). When there is no obvious choice of bin widths, reporting on varying bin widths can be helpful.

**Fig. 2:**
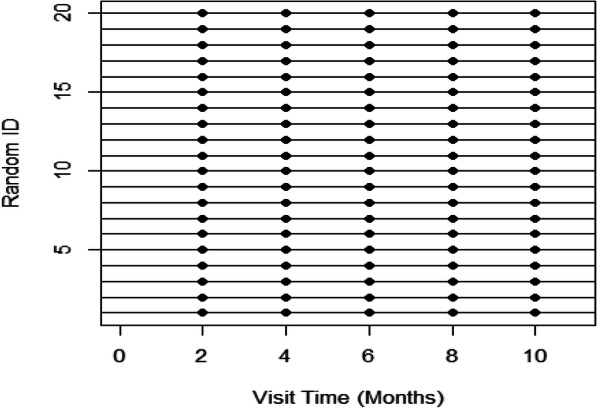
The visit timings for a random subset of 20 individuals from a perfect repeated measures simulated dataset with 100 observations

In perfect repeated measures, all individuals have 1 visit in a bin (regardless of bin width) and no individuals have 0 or > 1 visits per bin. Thus the proportion of individuals with 0 or > 1 visits per bin are 0 and the proportion of individuals with 1 visit per bin is 1. Figure 2 illustrates the visit timings for a random subset of 20 individuals from a perfect repeated measures simulated dataset with 100 observations and five pre-specified visit times (2, 4, 6, 8, 10 months). As the levels of missingness increase, the proportion of individuals with 0 visits per bin increases. As irregularity increases, the proportion of individuals with > 1 visit per bin increases.

The R code for plotting visiting patterns for a random subset of individuals and the mean proportions of individuals with 0, 1, and > 1 visits per bin uses the “IrregLong” package in CRAN [12] and is presented in the Appendix.

#### No pre-specified visit times

**Fig. 3:**
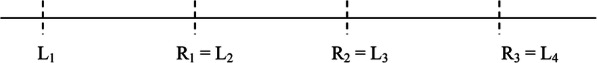
Specifying bin widths for no pre-specified visit times

We construct adjacent bins across the entire study period. Bin widths can be determined by clinical context or known visiting patterns (e.g. fewer visits later on in follow-up could be accommodated by wider bins). The j^th^ bin is given by the interval (L_j_, R_j_), where L_j_ and R_j_ are chosen to specify the left boundary and right boundary of the j^th^ bin respectively (Fig. 3).

The mean proportions of individuals with 0, 1, and > 1 visits per bin can be obtained by varying the number of bins (as the number of bins increases, bin widths decrease). These values can be used to judge the extent of irregularity by assessing whether or not they are consistent with values that would result from repeated measures. The larger the disparity of these values from repeated measures values suggests the greater the extent of irregularity. To evaluate this, the first step is to plot the mean proportions of individuals with 0, 1, and > 1 visits per bin as a function of bin width. The next step is to identify the bin width that yields the largest mean proportion of individuals with 1 visit per bin (i.e. in perfect repeated measures, all individuals have 1 visit per bin). At this bin width, determine if either the mean proportions of individuals with 0 or > 1 visits per bin are 0. If the mean proportion of individuals with > 1 visit per bin is not 0, this indicates a degree of irregularity. If the mean proportion of individuals with > 1 visit per bin is 0 and the mean proportion of individuals with 0 visits per bin is not 0, this suggests the data can be viewed as repeated measures with missingness. This comparison can be supplemented by identifying the largest bin width such that the mean proportion of individuals with > 1 visit per bin is 0, and evaluating whether the mean proportions of individuals with 0 and 1 visits per bin are 0. If at the largest such bin width, the mean proportion of individuals with 0 visits per bin is 0 and the mean proportion of individuals with 1 visit per bin is not 0, this suggests the data can be treated as repeated measures.

#### Censoring

Both left and right censoring should be considered when using bins to explore visit irregularity. Individuals may enter the study after the first pre-specified visit time, and the dataset may be closed before they have the opportunity to attend all the follow-up visits. In cases where censoring is administrative and unlikely to lead to bias, we may wish to measure irregularity separately from censoring. This can be done by specifying an “at-risk” set of individuals for each bin (i.e. individuals who are under follow-up for all times in the bin) then using just these individuals to estimate the proportions of 0, 1, and > 1 visits per bin. Individuals who are lost to follow-up (rather than administratively censored) can still be at-risk beyond their last visit. However, individuals should not be considered in calculations for bins representing times before they entered the study or after the dataset was closed.

## Results

### Pre-specified visit times: TARGet kids!

**Fig. 4:**
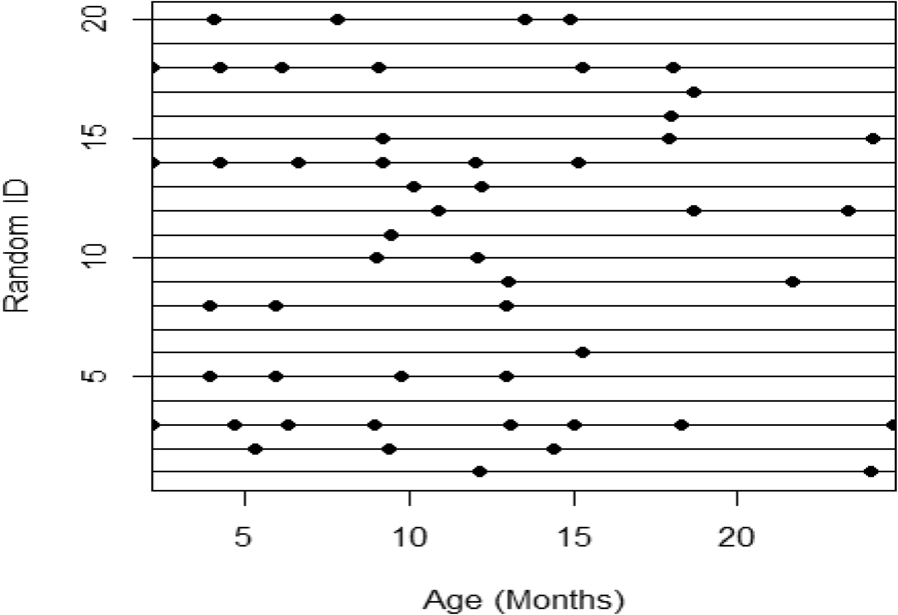
The age at visit (months) for a random subset of 20 individuals from the TARGet Kids! cohort

The study comprised of 6470 individuals with a median follow-up of 5.32 years. The years of recruitment ranged from 2008 to 2015. Data from well-child visits and sick visits were used to assess whether the data resembled repeated measures. Visits from all 6470 individuals were included in bin calculations, and Fig. 4 displays the age at each visit for a random subset of 20 individuals.

**Fig. 5:**
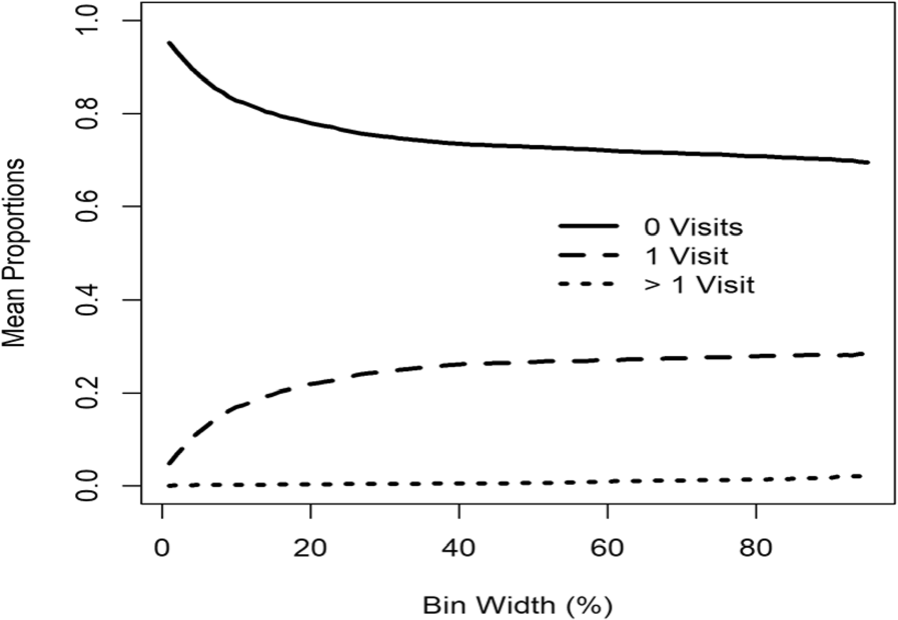
The mean proportions of individuals with 0, 1, and > 1 visits per bin as bin width varies from 1 to 95% of the gap between well-child visits for the TARGet Kids! cohort

All bins were anchored on the ages of well-child visits and the left side of each bin was fixed at 5% of the gap between successive well-child visit ages (since visits could not occur too early) and the right side of each bin was varied from 1 to 95% of the gap. Figure 5 illustrates the mean proportions of individuals with 0, 1, and > 1 visits per bin across varying bin widths. The mean proportions of individuals with 0 visits per bin were above 0.67 for all bin widths while the mean proportions of individuals with > 1 visit per bin were below 0.03. These values suggest that individuals mostly visit according to suggested visit times. The pattern is similar to repeated measures subject to missingness.

### No pre-specified visit times: cSLE study

**Fig. 6:**
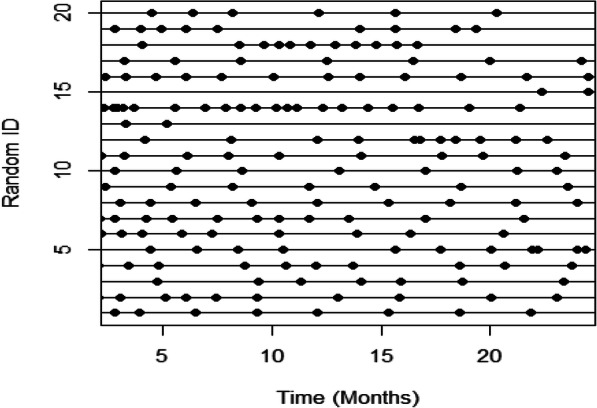
The visit timings for a random subset of 20 patients from the cSLE study

The study size was 473 individuals with a median duration of follow-up of 5.44 years (total duration of follow-up was 2666 patient-years). Figure 6 illustrates visit timings for a random subset of 20 individuals. Visit schedules highly varied and were personalized with few individuals having similar visit patterns.

**Fig. 7:**
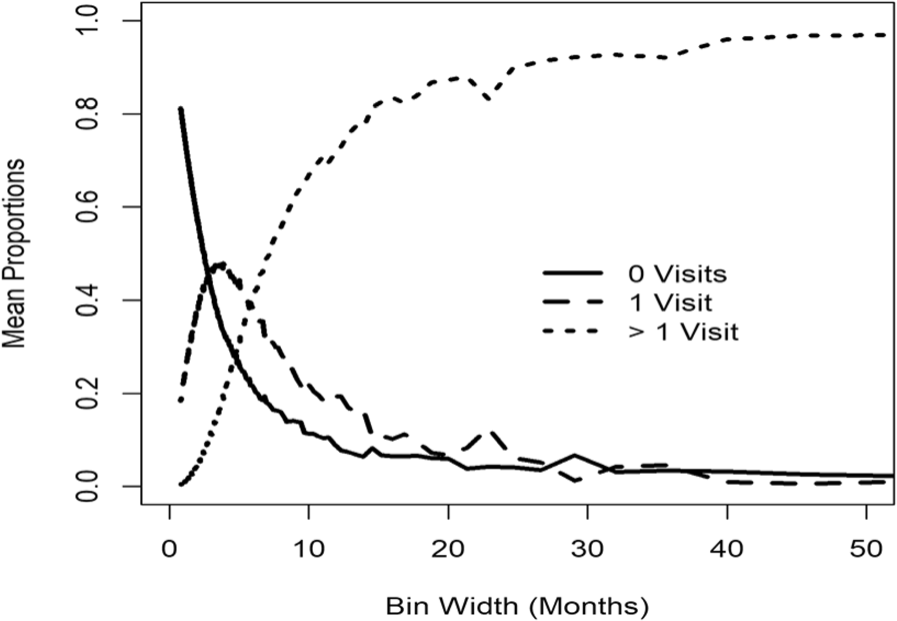
The mean proportions of individuals with 0, 1, and > 1 visits per bin for the cSLE study

To determine the extent of visit irregularity, the entire study period was split into adjacent and equally-sized bins and the number of bins was varied. Figure 7 shows the mean proportions of individuals with 0, 1, and > 1 visits per bin across bin widths. When the disease is controlled, individuals are recommended to visit every 6 months, and if their disease status worsens they visit more frequently. For bin widths of 6 months, the mean proportion of individuals with > 1 visit per bin was 0.39, the mean proportion of individuals with 1 visit per bin was 0.39, and the mean proportion of individuals with 0 visits per bin was 0.22. Although individuals were expected to visit at least once every 6 months, 22% of individuals on average had 0 visits when using this interval. The mean proportions of individuals with 1 visit per bin had a maximum value of 0.48 corresponding to bin widths of 3.52 months (the mean proportion of individuals with > 1 visit per bin was 0.15, and the mean proportion of individuals with 0 visits per bin was 0.37). For smaller bin widths of 0.82 months, the mean proportion of individuals with > 1 visit per bin was 0.004, the mean proportion of individuals with 0 visits per bin was 0.81, and the mean proportion of individuals with 1 visit per bin was 0.19. There were no bin widths that were consistent with repeated measures because even when the mean proportions of individuals with 1 visit per bin was maximized, 52% of individuals on average had > 1 or 0 visits per bin, and when bin widths were small enough such that the mean proportion of individuals with > 1 visit per bin was almost 0, 82% of individuals on average did not contribute data because they had 0 visits per bin. These results suggest individually-driven irregular visits, and therefore the extent of irregularity needs to be considered in analyses of the disease trajectory.

## Analyzing irregular visit processes

There are several methods which can accommodate irregular visit processes, but they make assumptions concerning the relationship between the outcome and irregularity [13]. It is important to consider the irregularity mechanism to ensure the validity of any chosen statistical approach for the outcome.

With missing data, judging whether data are missing completely at random (MCAR) or missing at random (MAR) is done by evaluating predictors of being observed at pre-specified occasions. This is typically done by comparing demographic and other available characteristics across the observed and unobserved groups using tables or logistic regression models [14]. With irregular data, the relationship between the outcome and visit process can be judged by identifying predictors of visit intensity.

### Visit processes

Determining the visit process is important because all methods make assumptions concerning the relationship between the visit and the outcome processes. Visit processes can be regular or irregular, and among irregular processes, the taxonomy for classifying missing data mechanisms has been extended to irregular visit processes [13]:

- **Visiting completely at random (VCAR):** Visit times are completely independent of the outcome process.
- **Visiting at random (VAR):** Given the observed history (outcomes, visits, covariates) up to time t, the visit process at time t, is independent of the outcome process.
- **Visiting not at random (VNAR):** Given the observed history (outcomes, visits, covariates) up to time t, the visit process is **not** independent of the outcome process at time t.

This classification scheme highlights the potential relationships between the outcome and visit processes over time and can be used to determine the appropriate analytic method for the outcome [13].

### Determining the visit process

To determine the visit process, it can be helpful to consider the study protocol. Some protocols pre-specify a common set of visit times (fixed visits), while others allow current patient status to determine future visit times such as: 1) a patient’s previously observed history (history-dependent), 2) physician-driven visits, or 3) self-determined or patient-driven visits.

If the protocol is adhered to perfectly, then history-dependent visits correspond to VAR. Physician-driven visits can also result in VAR provided that all the information that the physician uses to decide the time of the next visit is recorded in the patient’s chart. Patient-driven visits may be VNAR because the underlying factors which influence future visits are usually not reported in advance. It is important to consider the extent of deviations from pre-specified visit times for fixed, history-dependent protocols and physician-driven visits because the visit process may be non-ignorable, especially if deviations are due to unobserved or unrecorded factors.

Although it is possible to distinguish between VAR and VCAR visits using recurrent event regression models, there is no way of distinguishing between VAR and VNAR visits. Any modelling assumptions should be judged carefully to avoid biased results on the outcome.

#### Distinguishing between VCAR and VAR: Modelling the visit process

Identifying predictors of visit intensity can be performed using recurrent event regression models. Techniques for analyzing recurrent event data are well established [15] and are applicable to irregular visits. Regression models for recurrent events characterize event rates over time by modelling the intensity function [16]. The intensity function is analogous to the hazard function in survival analysis in the sense that it can be thought of as the instantaneous probability of observing an event by time t, conditional on a subject’s observed history.

One of the more commonly used intensity regression models is the Andersen-Gill model [17], which is an extension of the Cox proportional-hazards regression model [18]. The Andersen-Gill model is quite flexible as it can include time-dependent factors and past observed outcomes as predictors of future event intensity. The Andersen-Gill model can be implemented in standard survival analysis software such as R 3.1.0 [19].

#### Application to the cSLE study

Exploration of visits using bins indicated irregularity, therefore the visit process must be addressed. The following analyses aimed to identify predictors to help distinguish between VCAR and VAR. This was done by fitting a Cox proportional hazards regression model using the Andersen-Gill formulation with age at visit as the time variable. Baseline characteristics included: age at diagnosis (years), sex, race (Caucasian, Black, Asian, and Other), number of American College of Rheumatology (ACR) criteria for SLE at diagnosis, the presence of lupus nephritis at baseline, and mortality. Time-varying predictors included: disease activity measured by the SLE disease activity index [20, 21], prednisone dose, anti-malarial medication, total organ damage as measured by the SLE damage index [20], bone damage, cardiovascular damage (acute myocardial infarction, cerebrovascular accidents, and myocardial failure), a composite score for use of significant immunosuppression (any use of azathioprine for major organ disease, cyclophosphamide, cyclosporine, tacrolimus), and major organ involvement (including cerebrovascular accidents, psychosis, lupus nephritis classes III to V, pulmonary hemorrhage, myocarditis, major organ vasculitis).

The time-varying predictors included in the visit model were lagged by 1 visit. Model selection was based on fitting a regression model with all available predictors, and subsequently retaining predictors with *P*-values < 0.05. Analysis used the “coxph” function in R version 3.1.0 [22]. Table 1 presents the model summary.

**Table 1:** The visit process model for the cSLE study

Characteristic	Time-Varying	Hazard Ratio	95% Hazard Ratio Confidence Limits		***P***-Value
Disease Activity	Yes	1.02	1.01	1.02	< 0.0001
Prednisone (mg)	Yes	1.02	1.01	1.02	< 0.0001
Composite Score for Significant Immuno-Suppression	Yes	1.07	1.03	1.11	0.0010
Ethnicity	No				
Asian Vs. Caucasian	–	1.10	1.05	1.15	< 0.0001
Black Vs. Caucasian	–	1.20	1.13	1.26	< 0.0001
Other Vs. Caucasian	–	1.08	1.02	1.14	0.0064
Age at Diagnosis (Years)	No	1.02	1.01	1.03	< 0.0001
Major Organ Involvement Version 1	No	1.07	1.03	1.11	0.0012

The model confirmed that visit intensity was positively associated with disease activity (hazard ratio = 1.02, 95% confidence interval: 1.01–1.02). As a result, any regression analyses on disease activity should incorporate the visit process to account for this association; see [23] for an application of inverse-intensity weighted generalized estimating equations to this data.

The R code for modelling the visit process using the Andersen-Gill formulation and estimating the inverse-intensity weights are provided in the Appendix.

## Discussion

This paper proposes novel visual measures for summarizing the extent of visit irregularity by dividing the time frame of interest into bins and counting the number of individuals with 0, 1, and > 1 visits per bin. For the TARGet Kids! study, the mean proportions of individuals with 0 visits per bin were above 0.67 while the mean proportions of individuals with > 1 visit per bin were below 0.03. This suggested repeated measures data subject to missingness, and thus reasons for why visits are missing should be explored. If investigators deem missingness to be non-informative, the desired longitudinal outcome can be analyzed using appropriate missing data techniques such as multiple imputation. For the cSLE study, visits were recommended to occur at least once every 6 months. For bin widths of 6 months, the mean proportion of individuals with > 1 visit per bin was 0.39 and the mean proportion of individuals with 1 visit per bin was 0.39. The mean proportion of individuals with 1 visit per bin was maximized at bin widths of 3.52 months with a value of 0.48 (the mean proportion of individuals with > 1 visit per bin was 0.15 at bin widths of 3.52 months). Semi-parametric regression analyses on visit intensity showed that higher disease activity was associated with more frequent visits, and therefore regression analyses on the outcome should account for the visit process.

Irregular longitudinal data is often mishandled in practice. For example, researchers who know repeated measures ANOVA cannot handle irregular data assume they cannot use the data at all, or can use data from scheduled visits only. The latter approach can protect from bias when the visit process is VNAR; however, it is inefficient when the visit process is VCAR or VAR as outcome information is discarded. Other researchers may be aware that certain methods for longitudinal data (e.g. generalized estimating equations, mixed models) will run on unbalanced visits but falsely assume that the results will be unbiased, so they neglect the visit process and risk biased results. In the cSLE study for example, this would result in bias because individuals visited more frequently when their disease status worsened, and thus an unadjusted GEE analysis risks overestimating the burden of disease.

Visit irregularity and missing data are related concepts; however, the timings of visits are rarely scrutinized [9] whereas exploring missing data is recommended practice (e.g. STROBE, CONSORT 2010) [5, 6]. For example, the STROBE guideline encourages the reporting missing data by “indicating the number of participants with missing data for each variable of interest” [5]. Furthermore, identifying predictors of missingness is also generally recommended, see [14] for an example of how this can be done. Similar to missing data techniques, our measures of irregularity count the number of individuals with 0, 1, and > 1 visits in each bin. Fitting a recurrent event regression model for the visit intensity to distinguish between VCAR and VAR is analogous to using logistic regression to identify predictors of missingness.

Judging the visit process is crucial to modelling the outcome; we have presented this in terms of determining whether the visit process is VAR or VCAR; however, this can also be viewed in terms of ignorability. In missing data analysis, Little and Rubin [24] defined ignorability as not needing to model the missing data mechanism (data is missing at random or missing completely at random) when performing likelihood inference on the outcome. Farewell et al [25] extended the concept of ignorability to irregular longitudinal data and showed that stability is a sufficient condition for ignorability. Stability requires the outcome at the j^th^ visit to be independent of any visit patterns conditional on the observed data up to the j^th^ visit. In the presence of ignorability, parametric analyses can ignore the visit process.

Modelling the outcome trajectory using a mixed effects regression model is biased if the visit process depends on past observed outcomes and the covariance between the repeated measures is not correctly specified [26]. Several strategies can handle informative visit processes more effectively. Two main semi-parametric approaches for incorporating the visit process are: jointly modelling the outcome and visit processes using shared random effects [27] and constructing generalized estimating equations where observations are weighted by the inverse of their visit intensity [1]. Each strategy relies on a set of assumptions concerning the relationship between the visit and outcome process in relation to covariates and prior visits and outcomes [13]. Since each strategy was developed for specific visit scenarios, no modelling strategy can accommodate all possible cases. Thus careful consideration of the visit process and study design should inform the chosen analytic method.

While our proposed measures of irregularity can help to distinguish between repeated measures and irregular data, the specification of bin widths is not always straightforward. Consulting with a clinician may help in such cases. For example, the left side of the bins for the TARGet Kids! study was fixed at 5% of the gap between successive visits because it was understood that well child visits cannot be billed if they occur too early and vaccinations are not administered before a child is a certain age. We have also illustrated that varying bin widths can shed light on the visit process.

With missing data, the proportions of missing values provide an easily interpreted score of how severe the problem is. It would be ideal to have a single number that can be used to indicate the extent of irregularity. We are currently investigating the area under the curve (AUC) obtained from plotting the mean proportions of individuals with 0 visits per bin against the mean proportions of individuals with > 1 visit per bin. The AUC is a single number that can be used to describe the extent of irregularity where larger values of the AUC would signify increasing irregularity.

## Conclusions

Describing the extent of irregularity is an important step in determining the correct analytic approach to modelling the outcome. Choosing to ignore irregularity and simply use a mixed effects model leads to bias when the observed history (e.g. past outcomes and visits etc.) is predictive of future visit intensity. Exploring visit irregularity is as important as exploring missing data, and our measures of the extent of irregularity can assist in selecting the appropriate methodology for handling the longitudinal outcome.

## Data Availability

The datasets used and/or analyzed during the current study are available from the corresponding author on reasonable request.

## Appendix

### R-code for Generating Visual Measures and Modelling the Visit Process

##########Using the IrregLong Package to Generate Visual Measures.

Library (IrregLong).

Library (Survival).

##########Plot Visit Timings for a Random Subset of n Individuals.

abacus.plot(n,time,id,data,tmin,tmax,xlab.abacus = “Time”,ylab.abacus = “Subject”,

pch.abacus = 16,col.abacus = 1).

##########Plot Mean Proportions of Individuals with 0, 1, and > 1 Visit per Bin.

extent.of.irregularity (data,time = “time”,id = “id”,scheduledtimes = NULL,

cutpoints = NULL,ncutpts = NULL,maxfu = NULL, plot = FALSE,legendx = NULL,legendy = NULL,

formula = NULL,tau = NULL).

##########Modelling the Visit Process.

##########Create an “event” Indicator Representing When a Visit Occurred.

data$event<−c (1).

##########Visit Process Model.

model1 < −coxph (Surv (time_stop, time, event) ~ agedx+factor (eth) + ….., data = data).

summary (model1).

##########Weights.

data$p1 < −predict (model1,newdata = data,type = “lp”).

data$weights<− 1/data$p1.

summary (data$weights).

##########Test PH Assumption.

model11 < −cox.zph (model1).

model11.

## Abbreviations

cSLE: Childhood-onset Systemic Lupus Erythematosus
MAR: Missing at random
MCAR: Missing completely at random
MNAR: Missing not at random
VAR: Visiting at random
VCAR: Visiting completely at random
VNAR: Visiting not at random
GEE: Generalized estimating equation
ANOVA: Analysis of variance
